# Enhancing the interpretability of transcription factor binding site prediction using attention mechanism

**DOI:** 10.1038/s41598-020-70218-4

**Published:** 2020-08-07

**Authors:** Sungjoon Park, Yookyung Koh, Hwisang Jeon, Hyunjae Kim, Yoonsun Yeo, Jaewoo Kang

**Affiliations:** 1 Department of Computer Science and Engineering, Korea University, Seoul, South Korea; 2 Interdisciplinary Graduate Program in Bioinformatics, Korea University, Seoul, South Korea

**Keywords:** Computational biology and bioinformatics, Gene regulatory networks, Machine learning

## Abstract

Transcription factors (TFs) regulate the gene expression of their target genes by binding to the regulatory sequences of target genes (e.g., promoters and enhancers). To fully understand gene regulatory mechanisms, it is crucial to decipher the relationships between TFs and DNA sequences. Moreover, studies such as GWAS and eQTL have verified that most disease-related variants exist in non-coding regions, and highlighted the necessity to identify such variants that cause diseases by interrupting TF binding mechanisms. To do this, it is necessary to build a prediction model that precisely predicts the binding relationships between TFs and DNA sequences. Recently, deep learning based models have been proposed and have shown competitive results on a transcription factor binding site prediction task. However, it is difficult to interpret the prediction results obtained from the previous models. In addition, the previous models assumed all the sequence regions in the input DNA sequence have the same importance for predicting TF-binding, although sequence regions containing TF-binding-associated signals such as TF-binding motifs should be captured more than other regions. To address these challenges, we propose TBiNet, an attention based interpretable deep neural network for predicting transcription factor binding sites. Using the attention mechanism, our method is able to assign more importance on the actual TF binding sites in the input DNA sequence. TBiNet outperforms the current state-of-the-art methods (DeepSea and DanQ) quantitatively in the TF-DNA binding prediction task. Moreover, TBiNet is more effective than the previous models in discovering known TF-binding motifs.

## Introduction

Recently, deep neural networks have been widely applied to many bioinformatics problems and obtained better performance than conventional statistical and machine learning methods^1–3^. Predicting the functions of biological sequences (e.g., DNA sequences, amino acid sequences) is a representative task to which deep neural networks are successfully applied^4^. There are two main advantages of applying deep neural networks to biological sequences. First, designed in an end-to-end fashion, deep neural networks can predict the targets from raw input sequences without using hand-crafted features. Second, deep neural networks can automatically learn biologically meaningful features such as sequence motifs which are recurring sequence patterns associated with biological functions.

DeepBind^5^, mainly based on Convolutional Neural Network (CNN), was the early developed model to predict protein binding sites using deep neural networks. Interestingly, DeepBind could automatically learn sequence motifs represented as kernels of a CNN layer. Inspired by DeepBind, DeeperBind^6^ was proposed as an improvement over DeepBind. DeeperBind uses Long Short-Term Memory (LSTM) network, which is a variant of Recurrent Neural Network (RNN)^7^. In^8^, the authors used DeepBind as the baseline model and evaluated various CNN architectures to measure their effects on improving the TF-DNA binding prediction task by using ChIP-seq data from the ENCODE project. KEGRU, based on bidirectional Gated Recurrent Unit (GRU) network, was developed to improve the performance of the TF binding site prediction^9^. DeepSea^10^ which predicts the functions of DNA sequences using deep neural networks was proposed. Similar to DeepBind, DeepSea is also based on CNN architecture. However, DeepSea is trained to predict the DNA functions in a multi-task way whereas DeepBind is trained in a single-task way. DeepSea outperforms gkmSVM^11^ which was used as the best performing method at that time. DeepSea has also shown that the kernels of a CNN layer automatically learn sequence motifs. The authors utilized the output probabilities of DeepSea as features to predict the functional impact of non-coding variants. The classifiers trained on the DeepSea output achieved higher classification performance than representative variant scoring methods such as CADD^12^, FunSeq2^13^ and GWAVA^14^. DanQ which is an improvement over DeepSea was developed by adding a bidirectional LSTM layer to a CNN layer allowing the model to learn the regulatory grammars of sequence motifs^15^. DanQ outperformed DeepSea in both DNA sequence function prediction and functional non-coding variant prioritization.

Although the performance of deep neural networks in the prediction of biological sequence functions and TF-DNA binding site prediction has significantly improved, there is still much room for improvement. DanQ is currently the best performing neural network model with a CNN-RNN architecture for predicting DNA functions. Basically, a CNN layer captures the spatial information of an input sequence, and the kernels in the CNN layer function as motif scanners. A RNN layer learns regulatory grammars from the scanned motif information. In DanQ, it is assumed that the hidden motif features have the same importance for each DNA sequence position. However, for both models, it is difficult to interpret the prediction results obtained from the deep neural networks.

Attention mechanism was successfully applied to many machine learning tasks such as computer vision and natural language processing^16–19^. For example, in a machine translation task, the attention mechanism allowed neural networks to learn which part of a source sentence it should focus on for better translation^16^. In an image captioning task which involves generating a caption for a given image, the attention mechanism enabled neural networks to focus more on the part of an input image that is related to the generated word^17^. Recently, the attention mechanism has been also applied in several tasks in bioinformatics^20–24^.

When predicting TF binding sites, TF binding motif information in the DNA sequence can be used as important signals for the prediction. Thus, it is better to assign importance on the sequence region containing the TF binding motifs when predicting TF binding sites. To make a neural network assign high weights on the region having rich motif signals and improve interpretability, we propose TBiNet, an attention based neural network for predicting transcription factor binding sites. We trained TBiNet on the ChIP-seq dataset from 690 ChIP-seq experiments. TBiNet outperforms DeepSea and DanQ in predicting TF-DNA binding. Also, TBiNet extracts known TF binding motifs more than twice as much as the current top performing method. In order to interpret the attention layer in TBiNet, we visualized the attention scores of TBiNet. We observed that TBiNet assigns high attention scores at the actual binding sites of target TFs.

## Background

In this section, we describe the following three components which our model is built upon: CNN, LSTM, and an attention mechanism.

### Convolutional neural network

Convolutional Neural Network (CNN) consists of convolution layers usually followed by pooling layers with trainable weights and biases. A convolution layer takes a grid-like input and performs convolution operation using kernels which extract spatial information from inputs. A pooling layer compresses the information and reduces the spatial size of the input representation. Generally, non-linear activation functions are placed between a convolution layer and a pooling layer to learn non-linear relations between features. CNN has been widely used for computer vision such as image recognition and classification^25, 26^. Also, there are many researches on TF-DNA binding prediction tasks. For example, DeepBind^5^ and DeepSea^10^ treated a one-hot encoded DNA sequence as a 1D image and applied CNN to that sequence^8^ systematically explored how different architectures of CNN can affect performance on the TF-DNA binding task. One advantage of applying CNN to the TF-DNA binding task is that kernels in a CNN can learn TF binding motifs.

### Recurrent neural network

Recurrent Neural Network (RNN) is another type of neural network and is usually applied to sequential data where each part of a data is dependent on the previous part of the data. Unlike CNN, RNN has a flexible architecture that can process variable lengths of input and output. However, the basic RNN architecture is incapable of learning long-term dependencies due to the vanishing gradient problem. To address this issue of RNN^7^, introduced Long Short-Term Memory (LSTM). LSTM consists of an input gate, output gate, and forget gate that allows the model to either reflect or forget the impact of input data at each time step. The development of LSTM has led neural networks (e.g., seq2seq model) trained on sequential data to be more successful, especially in natural language processing^27^. Meanwhile, LSTM is also widely used in the bioinformatics domain, where data often contains sequential information such as DNA or amino acid sequences. Adding an LSTM layer to CNN-based models such as DeeperBind^6^ and DanQ^15^ helps improve their performance. Especially, DanQ uses BiLSTM which is a variant of LSTM, where inputs are fed once at the beginning of a sequence and once at the end of the sequence.

### Attention mechanism

Attention mechanism can assign different weight scores to each fragment of an input sequence to focus on more important fragments when generating outputs. With the attention scores, we can determine which part of the input is important to generate outputs. In^16^, the authors proposed a neural network with the attention mechanism for machine translation. The neural network was based on an encoder-decoder architecture. In this architecture, the attention scores between words in encoded sentences and each word in decoded sentences were computed.

The score $e_{ij}$ which indicates the alignment between the *j*-th word in the encoded sentence and the *i*-th word in the decoded sentence, is obtained by Eq. (1)

1 $$\begin{aligned} e_{ij} = a(s_{i-1}, h_j) \end{aligned}$$

where $h_j$ is the *j*-th hidden feature vector of the encoder, $s_i$ is the *i*-th hidden vector of the decoder. Although *a* can be defined in various ways, usually a simple feed-forward neural network or dot product is used. The score $e_{ij}$ is converted to a normalized weight ($\mathbf{a }_{ij}$) by applying the softmax function. Then, ($c_i$), the context vector of *i* is obtained by the weighted sum of the encoded feature vectors and their attention scores, as denoted in Eqs. (2) and (3)

2 $$\begin{aligned} c_{i}&=   \sum _{j=1}^t \mathbf{a }_{ij} h_j \end{aligned}$$

3 $$\begin{aligned} \mathbf{a }_{ij}&=   \frac{\text {exp}(e_{ij})}{\sum _{k=1}^{t} \text {exp}(e_{ik})} \end{aligned}$$

where *t* is the sequence length of the input.

## Methods

In this section, we explain the dataset used in this work and how we designed TBiNet in details.

### Dataset

ENCODE (Encyclopedia of DNA Elements) provides TF-DNA binding data analyzed by the ChIP-seq method^28^. For our experiments, we used the same dataset as used in DeepSea and DanQ which contains 690 ChIP-seq experiments from ENCODE. The preprocessing was done in DeepSea study. The preprocessed ChIP-seq dataset consists of 4,863,024 samples where each sample consists of a DNA sequence-target vector pair. A DNA sequence is represented as a 1,000 $\times$ 4 one-hot encoded matrix where each column corresponds to A, G, C, and T, respectively. A target vector is represented as a 690 $\times$1 binary vector where each element of the vector corresponds to one experiment out of the 690 ChIP-seq experiments. A ChIP-seq experiment can be represented as a TF-cell line pair (e.g., HepG2-p300). 172 TFs and 92 cell lines were included in the 690 ChIP-seq experiments. Input DNA sequence data was generated using the GRCh37 reference genome sequence. First, the entire sequence from the reference sequence is divided into 200 bp bins that do not overlap. For each task of a ChIP-seq experiment, if more than half of the given 200 bp bin belongs to a peak, the task is labeled as positive; otherwise, it is labeled as negative. Thus, 690 labels are assigned to each 200 bp bin. After labeling, 400 bp reference sequences are attached to both sides (left and right) of the 200 bp bin to create 1,000 bp input sequences.

Table 1 shows the statistics of the ChIP-seq dataset. The dataset is divided into the training, validation, and test sets. 4,000 samples from chromosome 7 were used as the validation set, 227,512 samples from chromosome 8 and 9 were used as the test set, and the rest were used as the training set. Reverse complement sequences were included in all the training, validation, and test sets; thus, there is a twofold increase in the number of samples. The list of 690 ChIP-seq experiments and positive sample information for each ChIP-seq experiment are described in Supplementary Table S1.

**Table 1:** Statistics of the ChIP-seq dataset.

Dataset	# Samples	Ratio (%)
Training	4,400,000	90.48
Validation	8,000	0.16
Test	455,024	9.36
Total	4,863,024	100.00

### Architecture of TBiNet

TBiNet consists of an input layer, CNN layer, attention layer, BiLSTM layer, fully connected layer and output layer. We designed TBiNet so that each kernel in the CNN layer captures a TF binding motif and the sequential information of the motif-related features in both directions in the BiLSTM layer. We use an attention layer to allow TBiNet to focus more on the positions containing TF binding motifs. For clarity, the notations are explained in Table 2.

**Table 2:** Notations of variables.

Notation	Description	Value
*S*	Sequence length	1,000
*l*	Kernel length	26
*h*	Total number of kernels	320
*w*	Maxpool window size	13
*t*	Maxpool output size	75
*x*	One-hot encoded input sequence	$x\in {\mathbb {R}}^{S\times 4}$
$\mathbf{F }$	Filter in CNN layer	$F\in {\mathbb {R}}^{l\times 4}$
$\mathbf{C }$	Output of CNN (Activation map)	$\mathbf{C }\in {\mathbb {R}}^{t\times h}$
$\mathbf{p }$	Key vector	$\mathbf{p } \in {\mathbb {R}}^{h}$
$\mathbf{a }$	Attention vector	$\mathbf{a } \in {\mathbb {R}}^{t}$
$\mathbf{C }_{scaled}$	Scaled $\mathbf{C }$ with Attention vector $\mathbf{a }$	$\mathbf{C } _{scaled} \in {\mathbb {R}}^{t \times h}$
$\mathbf{L }$	Output of BiLSTM	$\mathbf{L }\in {\mathbb {R}}^{t \times 2h}$
*z*	Output of the fully connected layer	$z \in {\mathbb {R}}^{695}$
$\hat{y}$	Output of TBiNet	$\hat{y} \in {\mathbb {R}}^{690}$

**Figure 1:**
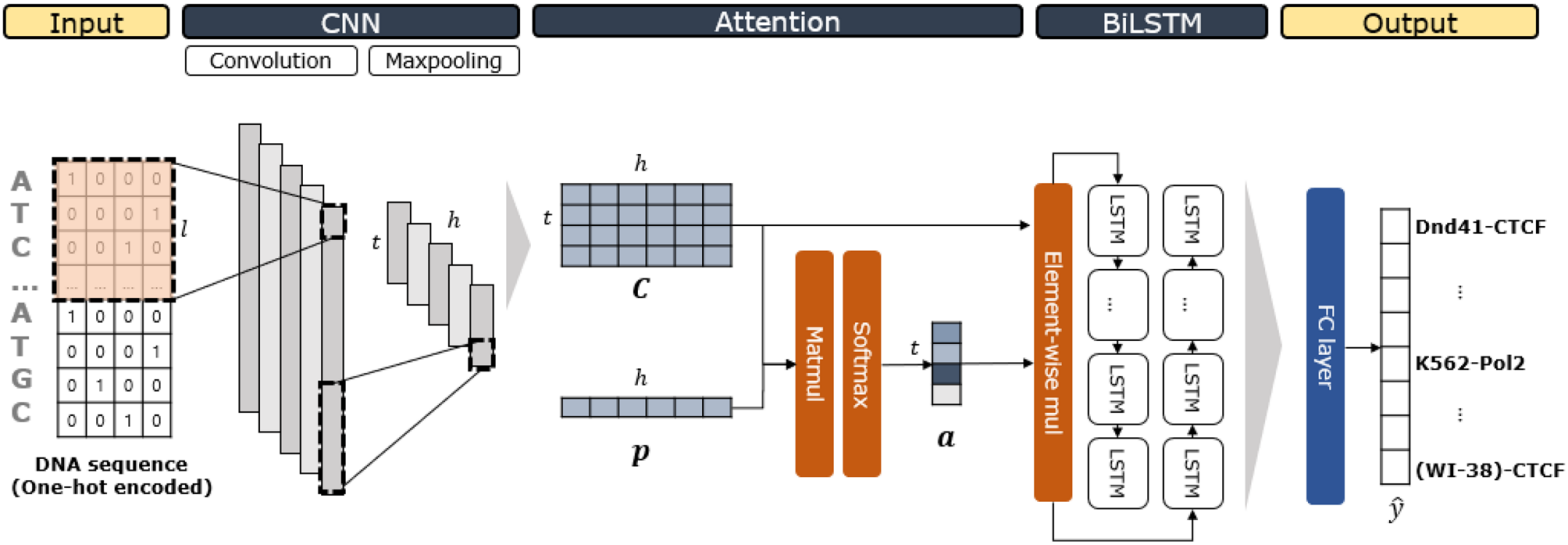
An illustration of TBiNet. An input DNA sequence is represented as a one-hot encoded $1{,}000\times 4$ matrix where each row corresponds to a sequence position and each column corresponds to a DNA nucleotide (‘A’, ‘G’, ‘C’, or ‘T’). Kernels in the CNN layer act as motif scanners. $\mathbf{C }$ matrix is the output of the convolution layer. $\mathbf{p }$ is a randomly initialized vector for attention (key vector). In the attention layer, matrix–vector multiplication is applied between $\mathbf{C }$ and $\mathbf{p }$. The output is then normalized by the softmax function which is referred to as an attention vector $\mathbf{a }$. Element-wise multiplication is applied between each column of $\mathbf{C }$ and $\mathbf{a }$. Then the matrix is passed to BiLSTM layer. The output of the BiLSTM layer is flattened and fed into a fully connected layer. A sigmoid function is applied in the output layer and $\hat{y}$ is the final output of the model which is a 690-dimensional vector where each dimension denotes a TF-cell line combination used for a ChIP-seq experiment.

Our model is illustrated in Fig. 1. An input DNA sequence is represented as a $S\times 4$ one-hot encoded matrix. *S* indicates the sequence length which is 1,000 and each of the 4 columns indicates a DNA nucleotide (‘A’, ‘G’, ‘C’, or ‘T’). The one-hot encoded input sequence is fed to a convolution layer. Kernel length is set to 26 and the number of kernels is 320. We applied rectifier activation after the convolutions; then the rectifier activation is followed by the max pooling layer keeping only the highest activation in a given window. Then, the activations of all kernels are concatenated at each position making an activation map. The following attention layer receives the activation map $\mathbf{C }$ as an input. Dot product between each row in the activation map and the $\mathbf{p }$ is applied. $\mathbf{p }$ is referred to as a key vector containing trainable parameters for the attention mechanism. We then applied softmax function to get attention vector $\mathbf{a }$. Next, we applied element-wise multiplication between *j*th column of $\mathbf{C }$ and $\mathbf{a }$. This scaled matrix $\mathbf{C }_{scaled}$ is fed into the following BiLSTM layer. The BiLSTM layer concatenates the outputs of LSTM for each direction (forward and backward) doubling the size of hidden dimension.

The output from BiLSTM layer is then flattened and fed into a fully connected layer followed by the output layer to generate the final output as a probability of the input sequence being the binding site of the TF in a given TF-cell line. The overall process of our model is given below.

4 $$\begin{aligned} c_i^k&=   \text {ReLU}(x_{i:i+l-1} *\mathbf{F }^k), 1\le i\le S-l+1 \end{aligned}$$

5 $$\begin{aligned} \text {ReLU}(x)&=   \text {max}(0, x) \end{aligned}$$

6 $$\begin{aligned} c_{pool}^k&=   \text {MaxPool}(\mathbf{c }^k) \end{aligned}$$

7 $$\begin{aligned} \mathbf{C }&=   [c_{pool}^1;c_{pool}^2;...;c_{pool}^h] \end{aligned}$$

where $c_i^k$ is an *i*-th convolution with *k*-th kernel $\mathbf{F }^k \in {\mathbb {R}}^{l\times 4}$, $x\in {\mathbb {R}}^{S\times 4}$ is a one-hot encoded input sequence, and ${*}$ denotes the convolution operation which is followed by a non-linear activation—Rectified Linear Unit (ReLU) defined as Eq. (5). The max-pooling operation extracts the maximum activation value of each kernel in a pre-specified window size *w*.

8 $$\begin{aligned} \mathbf{a }&=   \text {softmax}( \mathbf{C } \mathbf{p }) \end{aligned}$$

9 $$\begin{aligned} (\mathbf{C }_{scaled})_{*,j}&=   \mathbf{C }_{*,j} \odot \mathbf{a } \end{aligned}$$

10 $$\begin{aligned} \mathbf{L }&=   \text {BiLSTM}(\mathbf{C }_{scaled}) \end{aligned}$$

11 $$\begin{aligned} \tilde{y}&=   \text {ReLU} (\mathbf{W }_1^\top (\text {flatten}(\mathbf{L }) + \mathbf{b }_1) \end{aligned}$$

12 $$\begin{aligned} \hat{y}&=   \sigma ( \mathbf{W }_2^\top \tilde{y} + \mathbf{b }_2) \end{aligned}$$

where $\mathbf{p }\in {\mathbb {R}}^{h}$ is a trainable vector and $\odot$ denotes an element-wise multiplication. $\mathbf{W }_1 \in {\mathbb {R}}^{2ht\times 695}$ ($\mathbf{b }_1 \in {\mathbb {R}}^{695}$) and $\mathbf{W }_2 \in \mathbb {R} ^{695\times 690}$ ($\mathbf{b }_2 \in {\mathbb {R}}^{690}$) denote trainable weights (biases) of the fully connected layers and output layer, respectively. $\sigma$ denotes a sigmoid function. The loss is computed in a multi-task fashion with the prediction output and the ground truth target using the binary cross-entropy loss function defined as Eq. (13)

13 $$\begin{aligned} Loss = -\frac{1}{N}\sum _{n=1}^{N}\left[ y_n \text {log} \hat{y}_n + (1-y_n) \text {log} (1-\hat{y}_n)\right] \end{aligned}$$

where *N* is the number of samples and $y_n$ is a target label.

Table 3 shows the comparison between our model and the two state-of-the-art methods (DeepSea and DanQ). DeepSea uses a CNN with three convolutional layers and two max-pooling layers. DanQ model uses a CNN with only one convolution layer and one max-pooling layer. In DanQ, a BiLSTM layer is applied to learn regulatory grammars. Both CNN and BiLSTM have been shown to be helpful in the TF-DNA binding prediction task. However, by adopting the attention mechanism, TBiNet could focus more on DNA sequence region containing TF binding motifs and thus improve its performance.

**Table 3:** Comparison of neural network architectures between TBiNet and baseline methods.

Model	CNN	RNN	Attention
DeepSea	O	–	–
DanQ	O	O	–
TBiNet	O	O	O

### Experiment settings

TBiNet was trained using the Adam optimizer^29^ with a learning rate of 0.001. We set the batch size to 100 to minimize the average loss from the binary cross-entropy loss function. The validation set was used to determine whether our model overfits with the training set. The number of training epoch was 18. Training was conducted for about 7.5 h per epoch on a NVIDIA TITAN X GPU.

## Results

In this section, we discuss how we evaluate and compare the performance of our model with that of baseline models. We visualize the motifs found by CNN kernels from TBiNet that are matched with known TF binding motifs. We also analyze the attention layer in TBiNet to understand how the attention mechanism helps improve the performance of TF binding site prediction.

### TBiNet outperforms the state-of-the-art models in TF-DNA binding prediction

To quantitatively evaluate the accuracy of TBiNet in predicting TF-DNA binding sites, we tested TBiNet on the dataset containing data from the 690 ChIP-seq experiments. We compared TBiNet with the two state-of-the-art models: DeepSea^10^ and DanQ^15^. Those models were trained with the same 690 ChIP-seq dataset.

We used the following two evaluation metrics for evaluating TF-DNA binding predictions: AUROC and AUPR. AUROC is the area under the ROC (Receiver Operating Characteristic) curve where the x-axis corresponds to the false positive rate and the y-axis corresponds to the true positive rate. AUPR is the area under the precision-recall curve where the x-axis corresponds to recall and the y-axis corresponds to precision.

Table 4 shows the average AUROC and AUPR scores from all 690 ChIP-seq experiments. TBiNet achieved the highest AUROC and AUPR scores on average. The difference in AUPR performance between TBiNet and DanQ is greater than AUROC performance difference. This is meaningful since AUPR is a more appropriate evaluation metric than AUROC when the dataset is imbalanced^30^.

**Table 4:** Quantitative evaluation of DeepSea, DanQ, and TBiNet: average AUROC and AUPR scores obtained in the 690 ChIP-seq experiments.

Model	AUROC	AUPR
DeepSea	0.9015	0.2485
DanQ	0.9316	0.2959
TBiNet	0.9473	0.3332

**Figure 2:**
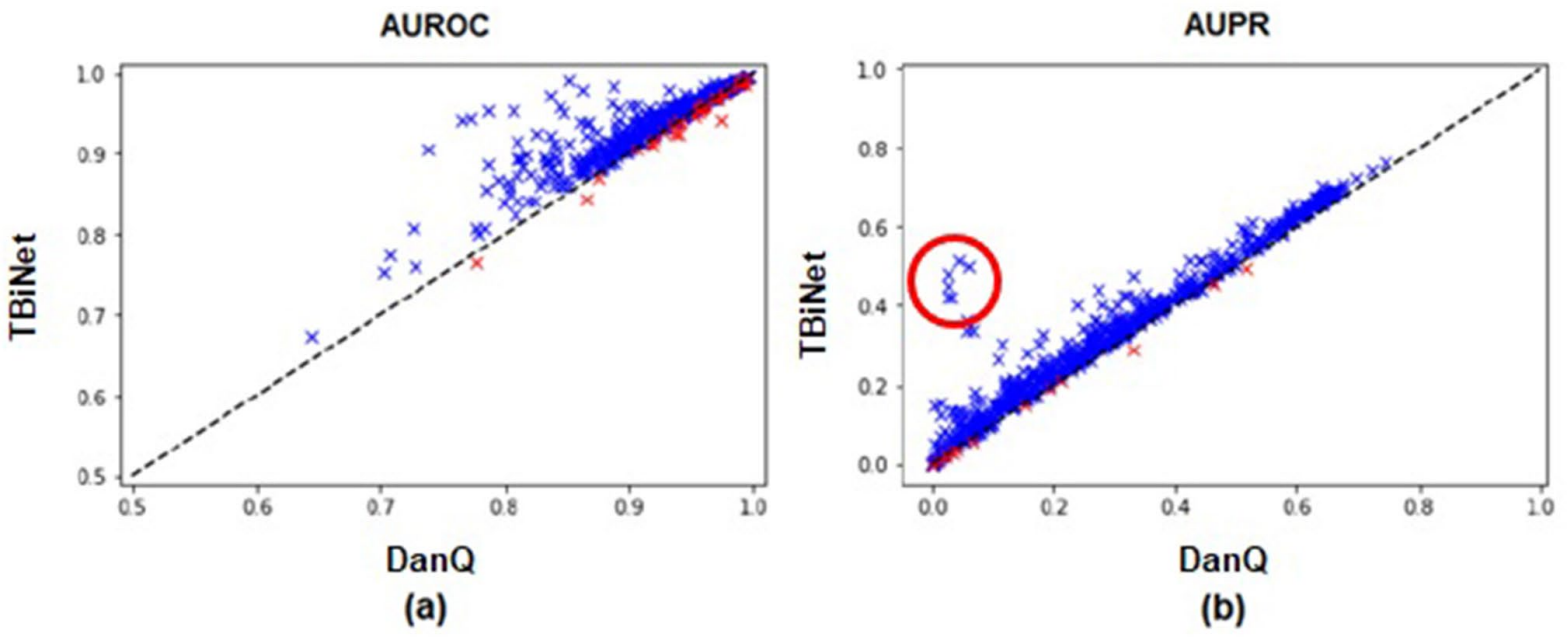
Scatter plot of AUROC and AUPR scores of DanQ and TBiNet. (**a**) AUROC scores for 690 tasks. The AUROC scores of DanQ are plotted on the x-axis and the AUROC scores of TBiNet are plotted on the y-axis. (**b**) Scatter plot of AUPR scores for 690 tasks. The x-axis corresponds to the AUPR scores of DanQ and the y-axis corresponds to the AUPR scores of TBiNet. Blue and red marks indicate the samples with a higher or lower score than DanQ, respectively. Red circled points in (**b**) are for further analysis.

Figure 2 shows the scatter plots where each point corresponds to the AUROC (or AUPR) score of a ChIP-seq experiment. The x-axis and y-axis indicate the score of DanQ and TBiNet, respectively. Compared with DanQ, TBiNet achieved higher AUROC scores for 653 out of the 690 (94.64%) experiments. In 675 out of the 690 (97.83%) experiments, TBiNet obtained AUPR higher scores than DanQ. We also evaluated the performance of TBiNet on the randomly shuffled dataset. TBiNet achieved the highest score on both AUROC and AUPR also on the randomly shuffled dataset (Supplementary Information Table S1).

**Figure 3:**
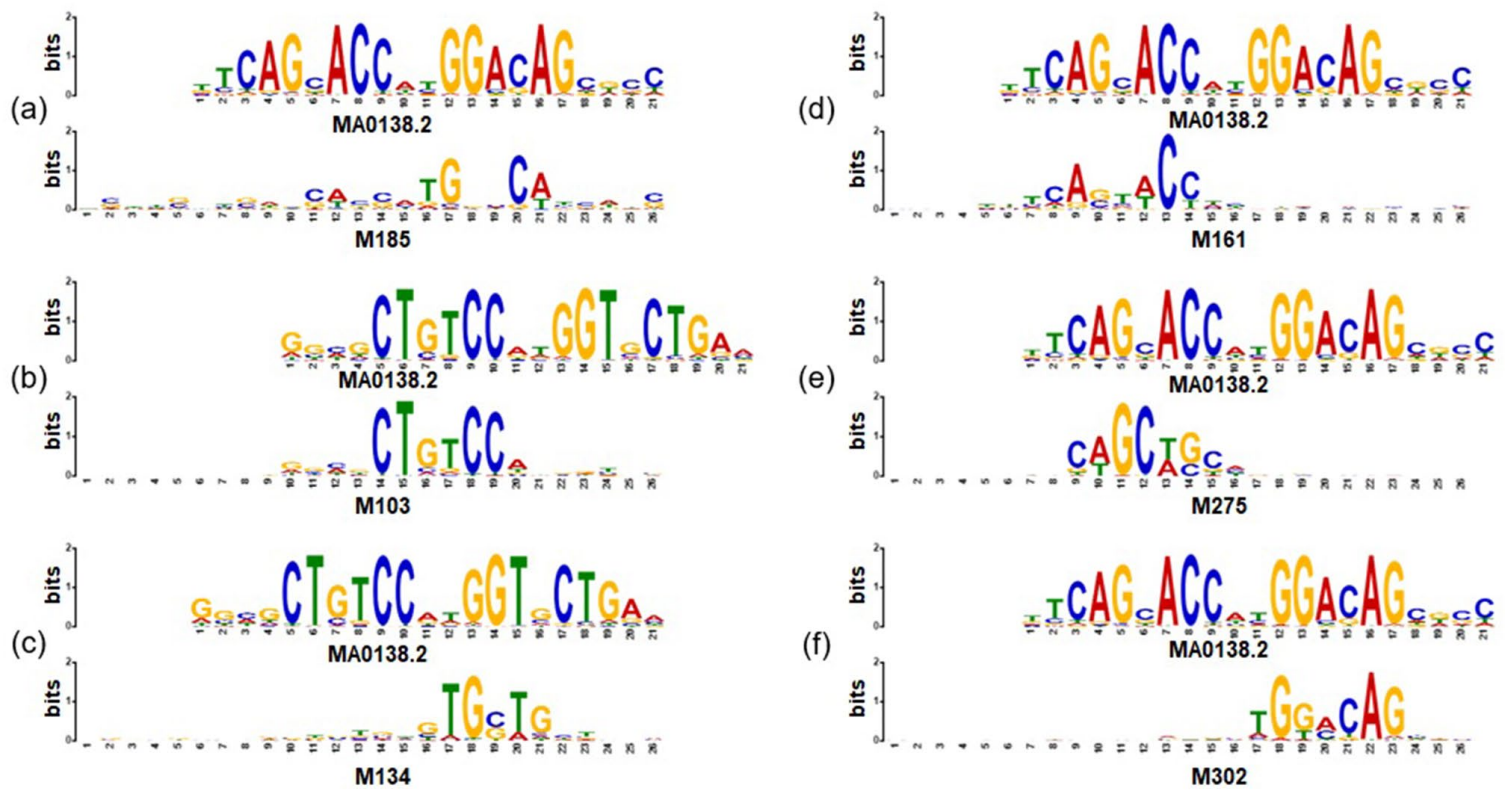
Visualization of CNN kernels resulting in exceptionally high AUPR scores. (**a**) A motif of NRSF for one CNN kernel from DanQ. (**b**–**f**) five CNN kernels from TBiNet that capture the same motif of NRSF. (**b**, **c**) Represent the reverse complement of the motif sequence. The top and bottom logo indicates the actual and predicted motif of NRSF respectively.

### TBiNet effectively extracts known TF binding motifs

A convolution operation allows kernels to extract spatial information from the input sequences. For example, if you have the input sequence ‘ATGCCA’ and you have a kernel with a length of 3, you will obtain a convolution value for each ‘ATG’, ‘TGC’, ‘GCC’, and ‘CCA.’ Then, an activation function such as a ReLU function is applied to those convolution values to add non-linearity to the model. If a kernel has the highest activation value at ‘ATG’, it can be inferred that ‘ATG’ is the most predictive feature for the task. In other words, after training a CNN to predict TF-DNA binding from DNA sequences, the kernels in the CNN layer can represent TF binding motifs. Many studies, such as^5, 15^ and^31^, have utilized the kernels in CNN layers for discovering TF binding motifs. We also tried to extract TF binding motifs from the kernels in the CNN layer.

To extract TF binding motifs from CNN kernels, we first feed all the input DNA sequences in the test set to TBiNet. We compute the activation values of all the kernels. For each kernel, the sequences of the position having the highest activation value (for only which that are greater than zero) are selected and collected for all the input DNA sequences. The collected DNA sequences are used to make a file in MEME motif format. The file contains position probability matrices (PPMs) where rows indicate DNA nucleotides or amino acids (here, DNA nucleotides) and columns indicate the position of each DNA nucleotide. An entry of a PPM is the probability of a DNA nucleotide appearing in the corresponding position.

**Figure 4:**
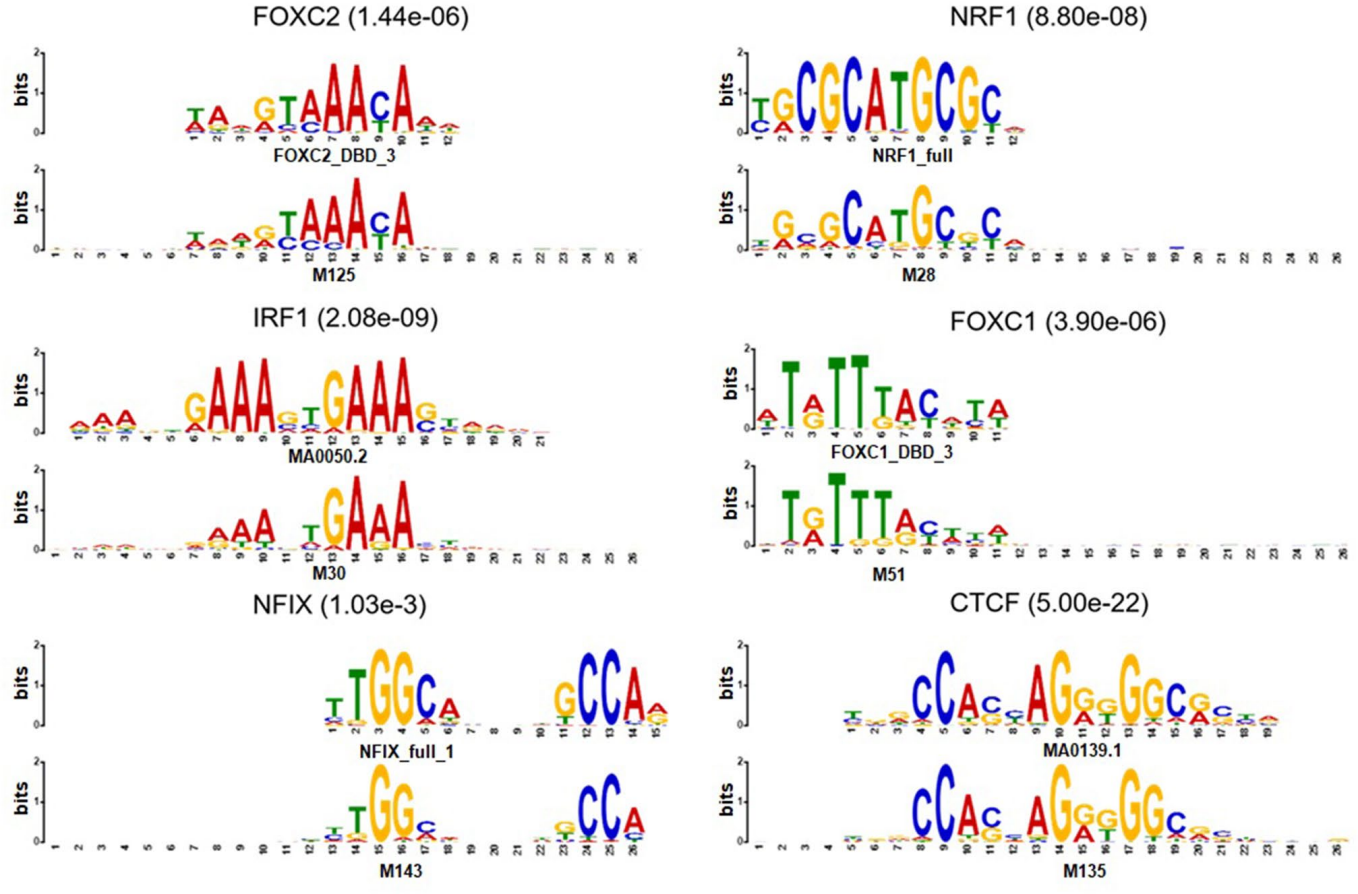
Examples of TF binding motifs extracted by TBiNet. The bottom sequence logo is obtained from TBiNet and the top sequence logo is similar to the motif from the reference motif database. The name of the transcription factor and the matching significance value (E-value) are shown above each figure.

Using the MEME formatted file, we ran TOMTOM^32^, which given the query motif, finds the significantly similar motifs in the reference motif databases. We used JASPAR 2016^33^, jolma2013^34^ and uniprobe^35^ as reference motif databases. We then counted the number of trained kernels in TBiNet that were similar to the known TF binding motifs. As a result, 142 out of 320 kernels of TBiNet were similar to known TF-binding motifs, which is more than that of DanQ (66 out of 320) and DeepSea (20 out of 320). As shown in Fig. 4, the TOMTOM results demonstrate that the trained kernels in TBiNet effectively represent known TF binding motifs.

To further analyze how TBiNet performed better than the previous model by effectively learning TF binding motifs, we focused on the ChIP-seq experiments that showed a significant improvement in AUPR score (red circled points in Fig. 2). It turned out that all top 5 ChIP-seq experiments were associated with NRSF. We then visualized the CNN kernels of TBiNet and DanQ as sequence logos (Fig. 3). It shows that five CNN kernels learned from TBiNet (Fig. 3b–f) were matched with the NRSF motif whereas only one CNN kernel from DanQ (Fig. 3a) was matched with the NRSF motif. Figure 3e matches to the front-end of the NRSF motif and Fig. 3f matches to the back-end of the motif. Similarly, Fig. 3b, c match to the front and back-end of the reverse complement of motif sequence, respectively. One explanation for this result is that TBiNet was able to capture more NRSF-related motifs in the CNN kernels than DanQ and those motif information was well aggregated in the following neural network layers. Moreover, as Fig. 3 shows, the CNN kernels of TBiNet capture motifs more clearly than that of DanQ.

**Figure 5:**
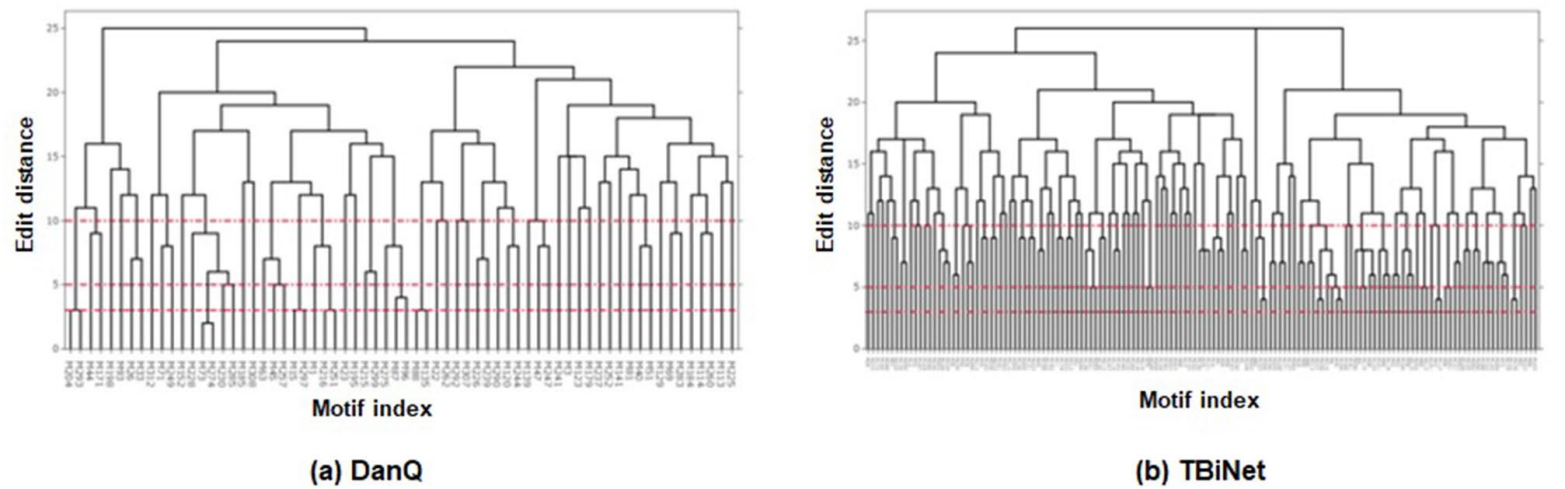
Dendrogram of clustered motifs by edit distance. x-axis indicates a motif index discovered by each model. y-axis indicates an edit distance. Red dotted lines are thresholds for removing redundant motifs.

We also examine whether our model extracts distinct motifs by eliminating redundant motifs from the discovered motifs. Figure 5 shows the dendrogram of clustered motifs from DanQ and TBiNet based on edit distance. Edit distance measures the similarity between DNA sequences. We apply edit distance to the consensus sequences of motifs which are obtained from TOMTOM. We assumed that redundant motifs have similar sequences. We set the threshold (*d*) of the edit distance as 3, 5 and 10 to filter distinct motifs only. As shown in Table 5, the distinct number of motifs changes with different distance thresholds. Though the number of distinct motifs decreases when threshold is 5 or larger, TBiNet still discovers more distinct motifs than DanQ across all thresholds.

**Table 5:** The number of distinct motifs filtered by edit distance.

Model	$\hbox {d}=0$	$\hbox {d}=3$	$\hbox {d}=5$	$\hbox {d}=10$	# of kernels
DanQ	66	61 ($\triangledown$ 7.58%)	58 ($\triangledown$ 12.1%)	40 ($\triangledown$ 39.4%)	320
TBiNet	142	142 (–)	136 ($\triangledown$ 4.23%)	91 ($\triangledown$ 35.9%)	320

*d* indicates the edit distance between motif sequences.

### Interpretation of the attention layer

**Figure 6:**
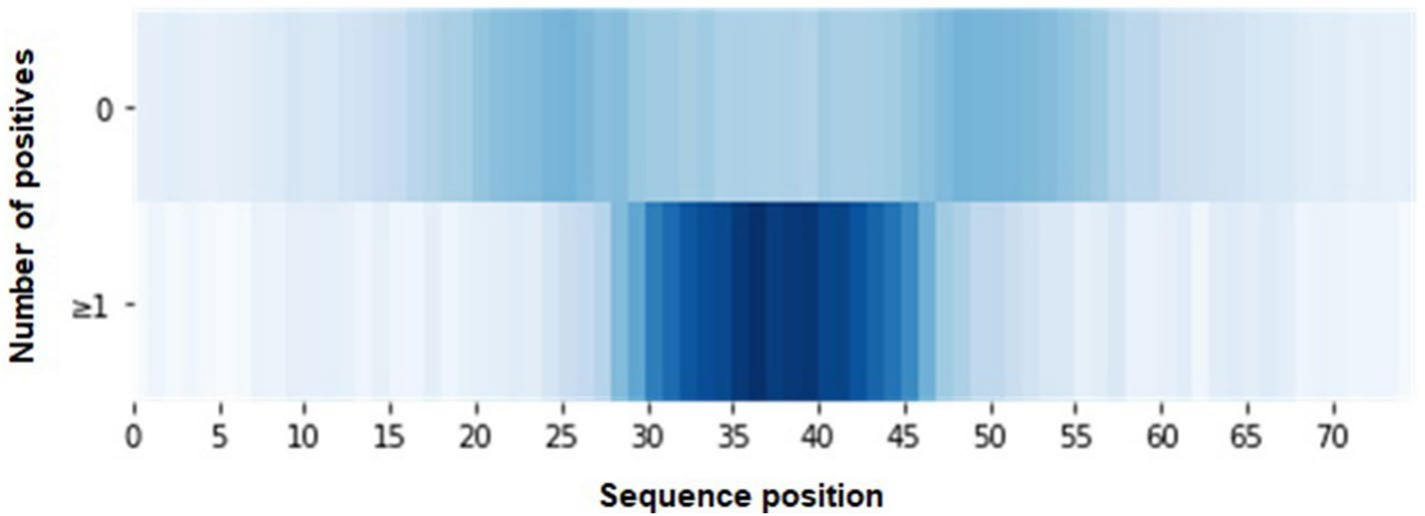
Visualization of attention scores. The top heatmap represents the averaged attention scores of the DNA sequences where none of TFs binds. The bottom heatmap is from the DNA sequences where at least one TF binds.

To understand how the attention has helped improve the performance and interpretability of TF-DNA binding prediction, we visualized the attention scores generated from TBiNet (Fig. 6). An attention vector ($\mathbf{a } \in {\mathbb {R}}^{75}$) is generated for each DNA sequence. An attention score in each dimension indicates the importance of each sequence position for predicting TF-DNA binding. Our hypothesis is that the sequence positions containing TF-binding motifs would have higher attention scores than other regions. Figure 6 represents the attention scores as heatmaps where the top heatmap indicates the averaged attention scores from the DNA sequences where TBiNet predicts as none of TFs binds. The bottom heatmap indicates the averaged attention scores from the DNA sequences where TBiNet predicts as at least one TF binds. It shows that the attention scores are significantly high in the central region, which is meaningful since true labels of TF binding sites were determined by the central region of the DNA sequence which belongs to the peak of TFs.

## Discussion

In this work, we proposed TBiNet which is an attention based deep neural network that predicts transcription factor (TF)-DNA binding from DNA sequence data. The kernels in CNN layer act as TF-binding motif scanners to capture TF binding motif information in input DNA sequences. The BiLSTM layer of TBiNet enables it to learn the regulatory grammars of the TF binding motifs obtained from the CNN layer. However, it is still assumed that all positions of motif features obtained from the CNN layer are equally important in prediction TF-DNA binding. We used the attention mechanism to allow TBiNet to focus more on important positions containing TF-binding motifs. With only additional 321 parameters, we could significantly improve the current state-of-art methods.

We first evaluated whether TBiNet can precisely predict TF-DNA binding. The ChIP-seq dataset from the ENCODE Project was used for the evaluation. The dataset contains data from 690 ChIP-seq experiments (i.e., 690 combinations of TF-cell lines). TBiNet outperformed DeepSea and DanQ in terms of AUROC and AUPR. TBiNet achieved significantly higher AUPR scores on the dataset on average, which is meaningful since the dataset is largely imbalanced. Since the regions of DNase I-hypersensitive sites (DHS) are similar to transcription factor binding sites, we also conducted an experiment that adds DHS labels into the target vector. Total 125 DHS labels were added into the target vector resulting in a 890 dimensional vector (DHS:125 + TF-binding:690). DHS labels were obtained from the DeepSea study. We observed that TBiNet significantly outperforms DeepSea and DanQ on the DHS-included dataset as well (Supplementary Information Table S2). In addition, TBiNet is able to extract more known TF-binding motifs than DanQ. Moreover, to understand how the attention mechanism helps improve the prediction performance and interpretability, we visualized the attention scores and showed that attention scores tend to be high at the actual transcription factor binding sites.

Although TBiNet has many advantages, we admit there is still room for improvement. TBiNet can predict the binding of TFs of a given DNA sequence in a certain cell line; however, it cannot predict binding for new TF-cell line combinations. Recently, TF binding prediction models such as FactorNet^31^ and TFImpute^36^ were developed and were able to predict the TF binding of new TF-cell line combinations. In future work, we plan to extend the architecture of TBiNet to utilize the features of cell lines (e.g., DNase I hypersensitive sites) or TFs (e.g., TF sequence embedding vectors), so that TBiNet can predict the binding of new TF-cell line combinations.

Using the attention mechanism, TBiNet outperformed the previous models in TF-DNA binding prediction. Also, TBiNet obtained better results in known TF binding motif extraction. We hope that TBiNet will be utilized for discovering novel TF binding motifs and identifying the effects of variants that arise in the binding sites of transcription factors.

## Supplementary information

Supplementary Information.

Supplementary Table S1.
