# Supplementary material for: Enhancing the interpretability of transcription factor binding site prediction using attention mechanism
Source: Sci Rep. 2020 Aug 7;10:13413. doi: 10.1038/s41598-020-70218-4 (PMC7414127; doi:10.1038/s41598-020-70218-4)
Supplement: Supplementary file 1 — Supplementary Information. [file 41598_2020_70218_MOESM1_ESM.pdf]

Supplementary Information

Enhancing the interpretability of transcription factor binding site prediction using attention mechanism

Sungjoon Park[1,†], Yookyung Koh[1,†], Hwisang Jeon[2,†], Hyunjae Kim[1], Yoonsun Yeo[1], and Jaewoo Kang[1,2,*]

1 .Department of Computer Science and Engineering, Korea University

2. Interdisciplinary Graduate Program in Bioinformatics, Korea University

*Corresponding author: kangj@korea.ac.kr

†these authors contributed equally to this work

Table S1 : AUROC/AUPR scores on the randomly shuffled test set.

	AUROC	AUPR
**TBiNet**	**0.9147**	**0.1774**
**DanQ**	0.8872	0.1513
**DeepSea**	0.8396	0.1185

Table S2 : AUROC/AUPR scores on the DNase I hypersensitive sites (DHS) added dataset. DHS labels were added in the target vectors resulting 815 dimensional vector (DHS: 125 + TF-binding: 690).

	AUROC	AUPR
**TBiNet**	**0.9414**	**0.3459**
**DanQ**	0.922	0.3024
**DeepSea**	0.8842	0.2446
